# Role of Autophagy and Apoptosis in the Postinfluenza Bacterial Pneumonia

**DOI:** 10.1155/2016/3801026

**Published:** 2016-06-08

**Authors:** Zhen Qin, Yuan Yang, Hongren Wang, Jun Luo, Xiaojun Huang, Jiangzhou You, Baoning Wang, Mingyuan Li

**Affiliations:** ^1^Department of Microbiology, West China School of Preclinical and Forensic Medicine, Sichuan University, Chengdu, Sichuan 610041, China; ^2^State Key Laboratory of Oral Diseases, Sichuan University, Chengdu, Sichuan 610041, China

## Abstract

The risk of influenza A virus (IAV) is more likely caused by secondary bacterial infections. During the past decades, a great amount of studies have been conducted on increased morbidity from secondary bacterial infections following influenza and provide an increasing number of explanations for the mechanisms underlying the infections. In this paper, we first review the recent research progress that IAV infection increased susceptibility to bacterial infection. We then propose an assumption that autophagy and apoptosis manipulation are beneficial to antagonize post-IAV bacterial infection and discuss the clinical significance.

## 1. Introduction

*(1) Influenza A Virus and Secondary Bacterial Infection*. It was reported that a pandemic influenza killed over 40 million people in 1918 [1, 2]. With the development of modern medicine and improvement of hygiene habits, the probability of influenza pandemic outbreak has been greatly decreased. However, influenza A virus (IAV), a negative-sense RNA virus with 8 separate segments in its genome [3], is very easy to mutate, which results in novel hemagglutinin (HA) production [4]. These mutations render IAV to invade easily population without immunity [5]. Studies have shown that it is the synergy between the viruses and bacteria that presents a great threat to public health while the viral infection alone rarely causes severe consequence [6–8]. Outcomes of influenza infection vary with age. Secondary bacterial infection has been considered as a key factor responsible for IAV death [7, 9]. Among them,* Streptococcus pneumoniae* (SP) and* Staphylococcus aureus* (SA) are the most commonly seen bacterial types [10, 11]. Additionally, the cytokine burst is another main cause of IAV-related death. Cytokine burst causes severe immunopathological damage to body [12] and also increases susceptibility to secondary bacterial infection [13].

The timing recognition of IAV and bacterial infection can be dated back to 1918 “Spanish flu.” At that time, pneumonia-associated deaths closely followed the outbreak of influenza. A set of bacteria such as SP and SA were incubated from these autopsies [14, 15]. Clinical data have suggested that secondary bacterial infection usually occurs 1-2 weeks after IAV infection and frequently causes IAV-related death [16, 17]. In exploring the pathogenic mechanisms underlying the excess pneumonia mortality after influenza infections, various animals were used as* in vivo* models to investigate viral-bacterial interaction. Among them, a murine model established by McCullers et al. preferably manifested the clinical characterizations [18, 19]. In their study, IAV and SP were used to infect mice at various time sequence or pathway, which produced an intriguing mortality difference. The mortality was 60% in mice infected with IAV and SP simultaneously. It was up to 100% when SP was inoculated 7 days after IAV infection. On contrast, mice challenged with SP 7 days before IAV survived 100%. Mice infected with either IAV or SP alone had mortalities of 35% or 15%, respectively. Studies from other labs also supported that influenza infection followed by bacterial challenge rendered the most severe outcomes [20–24]. Thus, these results suggest IAV infection facilitates secondary SP infection.

**Figure 1:**
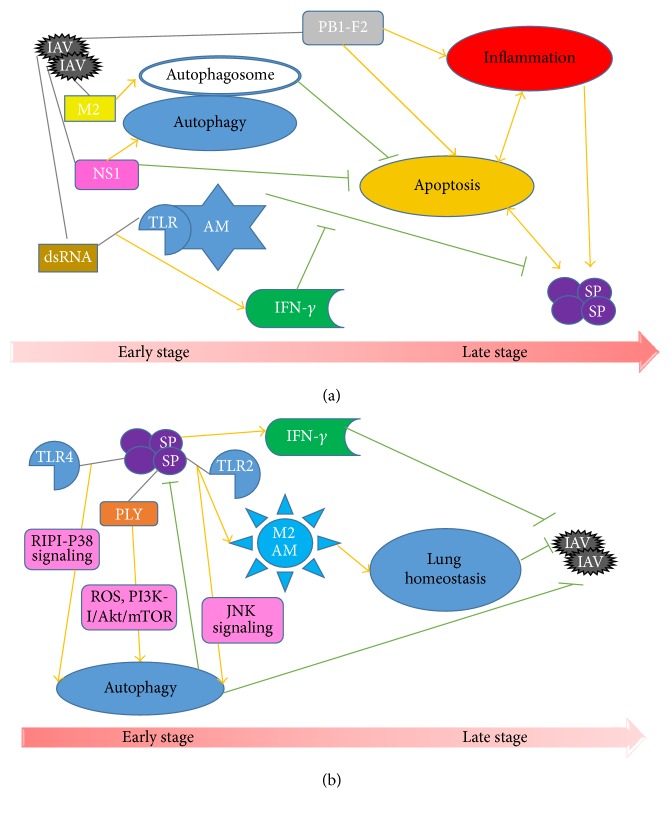
Autophagy and apoptosis in the early and late stage of IAV-SP mixed infection. In the figure, the orange arrows represent links of stimulation, whereas the green bars correspond to inhibitory links. (a) Autophagy and apoptosis seem to act as sequential events after IAV infection. NS1 plays a critical role in regulating IAV-induced autophagy and apoptosis. M2 is necessary for the formation of autophagosomes. The latter delays the development of apoptosis. PB1-F2 induces inflammatory response, which has a mutual promotion with apoptosis. PB1-F2 of PR8 can cause apoptosis of monocytes. Both apoptosis and inflammation contribute to secondary bacterial infection. Recognition of viral dsRNA by TLR of alveolar macrophages promotes IFN-*γ* production, which prevents macrophages from clearing bacteria (such as SP). (b) SP triggers autophagy by interacting with TLR4 and TLR2 through RIPI-P38 signaling and JNK signaling, respectively. By the interaction of SP and TLR2, M2 alveolar macrophages (M2 AM) polarized from monocytes help maintain the lung homeostasis. PLY stimulate autophagy through ROS hypergeneration and PI3K-I/Akt/mTOR pathway. In addition, SP causes IFN-*γ* increase, together with autophagy and the maintenance of lung homeostasis, which alleviates subsequent IAV infection.

*(2) Prevailing Mechanisms of IAV Facilitating Hosts to Bacterial Pneumonia*. Multiple factors are involved in virally bacterial pneumonia. Of them, respiratory epithelial damage is considered as the most classical one since the 1918 pandemic; that is, IAV incursion exposes the binding sites to bacteria [19, 25, 26]. This has been further proved by increasing number of pathological researches involving viral and bacterial infection [27–29]. In addition, the recognition of IAV by toll-like receptors (TLRs) increased interferons (IFNs) secretion. The latter suppressed the functions of macrophages and neutrophils and led to the failure of bacterial clearance [30, 31]. To observe the dynamics of timing and sequential infection between IAV and SP, Shrestha et al. developed a mathematical model to simulate these processes [32]. Consistent with results of McCullers and Rehg that pneumococcal challenge 7 days after IAV infection leads to the most severe disease state and rapid death [19], Shrestha et al. found that SP infection 4–6 days after influenza infection was the most efficient model to cause invasive pneumonia [32]. One main cause for the delayed SP infection is believed to be due to the inhibition of alveolar macrophages by IFN-*γ*, a product after IAV recognition by TLRs (Figure 1(a)) [33]. Other IAV-related factors promoting bacterial susceptibility include mucociliary dysfunction [34], bacterial receptors expressing on epithelial cells [35, 36], cytokines increasing vascular permeability (e.g., interleukin-16 (IL-6) and tumor necrosis factor-*α* (TNF-*α*)) [37], cytokines inhibiting early immune response (e.g., IL-35, IL-17, and IL-10) [30, 38, 39]. Investigations involving classical innate and adaptive immune responses on IAV infection and its sequelae have been well documented (as reviewed in [25, 40–42]). However, the role of apoptosis and autophagy is less reported, especially in IAV-related bacterial pneumonia.

## 2. Role of Autophagy and Apoptosis in Viral-Bacterial Interaction

Interestingly, recent studies have shown that autophagy, an evolutionarily conserved cellular pathway existing ubiquitously in eukaryotes to degrade unwanted cytoplasmic materials such as long-lived proteins and organelles under stressed conditions like nutrition deprivation and hypoxia [43, 44] is involved in IAV infection [45, 46]. Autophagy was initially found to protect against microbial invasion. Some viruses, such as influenza viruses, have evolved to subvert this mechanism for their own benefit [47]. Zhou et al. first reported that autophagy was related to IAV replication and that virus yield was decreased by autophagy suppression [45]. They also reported that viral titer was decreased by enhanced autophagy at another study [48]. Additionally, autophagy has been considered as a new programmed cell death way. Evidences showed that cell death induced by H5N1 is predominantly autophagic rather than apoptotic. Autophagic cell death was considered as a main factor causing severe lung injury in H5N1-infected mice. This injury can be ameliorated by suppressing autophagy but not apoptosis [49]. These data suggest that autophagy, to a certain extent, is involved in H5N1-related cell death both* in vitro* and* in vivo*. Nevertheless, in some pathologic situation, excessive autophagy might also lead to cell death through apoptosis (as reviewed in [50]). Although autophagic cell death was more likely to be induced in highly pathogenic strains [49, 51], whether autophagy is a way of executing cell death or cell death is accompanied by autophagy remain controversial [50, 52].

Apoptosis, classified as type I programmed cell death, is generally characterized by nuclear fragmentation, chromatin condensation, cell shrinkage, plasma membrane blebbing, and intact cell membrane [53–55]. Relationship between IAV and apoptosis was early studied* in vitro* and* in vivo* [56–58]. Apoptosis was originally thought not to cause inflammation. Later studies showed that IAV induced caspase-1 and caspase-3, which proteolytically processed IL-1*β* and IL-18, and subsequently indirectly caused inflammatory responses [59], including in respiratory epithelial cells and leucocytes [60]. By recognizing viral RNA, members of nucleotide-binding domain and leucine-rich-repeat-containing (NLRs) family such as cryopyrin assemble inflammasomes to activate caspase-1 and then increase IL-1*β* and IL-18 secretion in macrophages [61, 62]. Thus, it is stimuli inducing apoptosis that determine whether apoptosis causes inflammatory response [63].

Many IAV proteins are involved in apoptosis, such as nucleoprotein (NP), matrix protein 1 (M1), matrix protein 2 (M2), nonstructural protein (NS1) [64], and PB1-F2 [65]. Fourteen years ago, in investigating an unknown antigenic peptide of IAV presented by CD8+ T lymphocytes, Chen et al. found a protein, PB1-F2, the eleventh viral protein encoded by the open reading frame (ORF) of PB1 gene. This strain specific protein is considered as one of the virulence factors contributing to the high pathogenicity of IAV, including the ability in promoting secondary bacterial infection by inducing cytokine storm [66, 67]. Thus, IAV tends to cause an inflammatory apoptosis.

### 2.1. Autophagy and Apoptosis Facilitates Secondary Bacterial Pneumonia after IAV Infection

#### 2.1.1. IAV Induces Autophagy

Several IAV proteins are involved in progress of viral infection promoting bacterial superinfection via the regulation of autophagy and apoptosis, in which autophagy and apoptosis appear to be sequential events (Figure 1(a)). NS1 protein was expressed at the early stage of IAV infection [68]. It was reported that NS1 protein indirectly promoted autophagy at the early stage of IAV infection through upregulating the synthesis of HA and M2 [69] and downregulating apoptosis to facilitate viral replication [70]. NS1 protein also positively regulated PI3K-Akt pathway to inhibit apoptosis in the early stage of IAV infection, while in the late stage, NS1 induced p53 dependent or independent pathways to activate apoptosis [71]. Besides, NS1 may suppress apoptosis partly by antagonizing IFN. Apoptosis was enhanced and accelerated in IFN-competent cells infected by NS1-depeleted variant (delNS1) but was delayed in IFN-deficient cells infected by wild-type or delNS1 strains [70]. Furthermore, NS1 also exerted antiapoptosis effect by blocking the recognition of recognizing double-stranded RNA (dsRNA) dependent protein kinase (PKR) to viral dsRNA [72–75]. Notably, apoptosis suppressive effect of NS1 is strain specific. NS1 exhibited apoptosis suppression in some strains, like H1N1, H3N2, and H7N7 [70, 76]. Conversely, in some strains, like H5N9 and H5N1, NS1 promoted apoptosis [77, 78]. M2 protein acted as a proton channel for viral uncoating after fusion in endosomes [3]. M2 protein is also found to be necessary for autophagosome formation. Silencing M2 expression resulted in significantly reduced autophagosome accumulation during IAV infection. However, M2 blocked IAV-induced fusion of autophagosome and lysosome via binding to Beclin-1 with its first 60 amino acids [79]. As autophagy deficient cells exhibited enhanced apoptosis, M2-mediated autophagosome accumulation is likely to decrease apoptosis [79]. On the whole, influenza virus provides environment and time for viral replication by inhibiting apoptosis and triggering autophagy at the early stage of infection.

#### 2.1.2. Autophagy in IAV Infection: Two Sides of a Coin

Autophagy is a doubled edge sword. On the one hand, autophagy is a prosurvival pathway in which it protects against various pathogenic invasions, including IAV [80]. The antiviral character of autophagy is mostly attributed to its function in adaptive immunity. One recent study showed that autophagy was essential for the maintenance of memory B cells against IAV infection* in vivo* [81]. In this study, Chen et al. demonstrated that mice with autophagy-related gene (Atg) 7 knockdown in B cells failed to produce secondary antibodies when they were infected with IAV again [81]. Similarly, autophagy displays a prosurvival role in effector CD8+ T cells during influenza infection. By using an inducible Atg5 knockout mouse system, Schlie et al. found that mice infected with IAV failed to recall a primary response peak, and the Atg5−/− CD8+ T cells exhibited feeble viability and upregulated P53 expression [82]. Additionally, a natural compound, pentagalloylglucose (PGG), which was reported to have anti-influenza activity [83], promoted autophagic flux via degradation of viral M2 protein, a protein that blocks the fusion of autophagosome and lysosome at enough high concentration, and subsequently caused the downregulation of several viral proteins, like NP, M1, HA, and M2 [84]. On the other hand, autophagy is beneficial to IAV replication and production. Zhou et al. reported in an* in vitro* experiment that autophagy was involved in IAV replication and autophagy suppression and decreased viral yield [45]. A large amount of studies showed that autophagic deficiency reduced IAV virulence [45, 51, 84–91]. It is noteworthy that IAV-induced autophagy is both strain and cell specific [46, 92]. In addition, autophagy is associated with influenza-induced inflammatory. For instance, TLR3 enhanced autophagy by dsRNA to promote the production of IFN and some other cytokines [93, 94]. Autophagy was also involved in the induction of IFN-*α* and CXCL10 in H9N2/G1 infected cells [46, 92]. Moreover, autophagy-mediated inflammatory response was also associated with nuclear factor-*κ*B (NF-*κ*B) and p38 mitogen-activated protein kinase (MAPK) signaling in H5N1 pseudovirus infection. This signaling in turn promoted the formation of autophagosomes, suggesting an important mechanism underlying H5N1-related hypercytokemia [95].

#### 2.1.3. IAV-Induced Apoptosis: More of a Foe Than a Friend

Apoptosis has dual characters as well as autophagy. Several proapoptotic factors definitely play an antiviral role. Recently, Chang et al. found that several avian influenza viruses induced early apoptosis in porcine alveolar macrophages, which inhibited viral replication and mitigated inflammation [96]. IL-24 was found to decrease IAV titer by activating TLR3 dependent apoptosis [97]. Although initial findings show that apoptosis is a host defensive mechanism against IAV infection [98], generally speaking, apoptosis is beneficial to viral replication, dissemination, and host immune cells kill. As such, apoptosis may serve as a contributor for secondary bacterial infection following influenza virus infection.

Firstly, IAV indeed triggers apoptosis through various mechanisms to damage host immunity ability. Some viral components, such as NS1, M2, PB1-F2, M1, NA (neuraminidase), NP, and dsRNA, are associated with apoptosis regulation. As described above, viral NS1 plays an antiapoptotic role in host immune response [69–75]. Combined with M2, these viral components regulate autophagy and apoptosis as sequential events [79]. PB1-F2, a viral protein encoded by an open reading frame of IAV PB1, is shown to induce apoptosis at the late stage of IAV infection via mitochondrial permeabilization in strain dependent and cell specific manners [65]. It is notable that only PB1-F2 produced by influenza A/Puerto Rico/8/34 (H1N1) (hereafter referred as to PR8), but not other strains, induced alveolar macrophages death rather than epithelial cells in the lung [65, 99, 100]. As a result, PB1-F2 interferes in viral and bacterial clearance and antigen presentation at the early stage. McAuley et al. introduced PB1-F2 protein of 1918 influenza H1N1 virus into PR8, resulting in a higher susceptibility to secondary bacterial pneumonia than wild-type PR8. Nonetheless, they also observed that PB1-F2 knockout variant resulted in lower mortality when followed by SP infection as compared to wild-type PR8, which expresses PB1-F2 with 87 amino acids [101], although both had similar viral loads in lung [66]. Recently, Yoshizumi et al. found that full-length PB1-F2 of highly pathogenic IAVs translocated into mitochondria via Tom40 channels and then impaired innate immune and contributed to symptomatic deterioration, while truncated PB1-F2 (lacking C-terminal region responsible for translocating into mitochondria) from low pathogenic IAVs was less harmful due to disability in translocating into mitochondria [102]. These findings indicate that PB1-F2 exerts the pathogenicity on postinfluenza bacterial infection more likely through other mechanisms rather than apoptosis. One of the most direct causes is excessive inflammation [103–105]. An* in vivo* experiment with different viral strains discovered that PB1-F2 was related to inflammatory infiltration of macrophages and neutrophils, hypertrophy of epithelial cells, and fibrin deposition [67]. Additionally, PB1-F2 induced inflammatory response by activating inflammasome [106], regulating NF-*κ*B and IKK*β* activity [107] and forming aggregates [108]. M1 and NA protein induced apoptosis by interacting with caspase-8 [109, 110] or activating tumor growth factor-*β* (TGF-*β*) [77, 111]. Human Clusterin (CLU) prevented intrinsic apoptosis pathway through binding to Bax, which interfered with viral NP protein [112]. dsRNA virus-mediated apoptosis was reported to be related to caspase-dependent pathway [113], PKR, TLR, retinoic acid-inducible gene (RIG), and other forms of signaling [114–117].

Secondly, many reports have shown that apoptosis inhibition decreases IAV pathogenicity. Herold et al. reported that macrophages, when recruited from peripheral blood to the lungs during IAV infection, released tumor necrosis factor-related apoptosis-inducing ligand (TRAIL) to induce apoptosis of alveolar epithelial cells and increase lung leakage and mortality, which in turn were rescued by blocking TRAIL signaling [118]. Additionally, Liu et al. found that caspase inhibitors decreased viral replication and release of certain kinds of proinflammatory cytokines and chemokines in IAV infected mast cells [119]. Jaworska et al. discovered that the interaction of host NLRX1 and viral PB1-F2 protein suppressed mitochondria-related apoptosis and enhanced macrophage function, which, as a result, mitigated viral replication, lung function disorder, and mortality [120]. Tran et al. used human whole-genome screen method to search for cell death related genes in IAV infection. USP47, TNF superfamily (TNFSF) 13, and TNFSF12-13 were identified as important components. Their depletion produced host protective effects [121].

As mentioned above, SP significantly aggravates IAV infection. To explore the role of apoptosis in postinfluenza SP pneumonia, Kosai et al. infected mice with IAV or SP alone or IAV 48 hours followed by SP. They found that apoptosis occurred earlier and more severe in mice infected with combination of IAV and SP than IAV or SP alone [122].

### 2.2. Preceding SP Infection Alleviates Onsets of Subsequent IAV Invasion: Role of Autophagy

#### 2.2.1. Preceding Bacterial Infection Protects Host from IAV Infection

A prior bacterial exposure may protect the host from adverse impacts of following IAV infection. This concern is mainly derived from study made by McCullers and Rehg. In their study, mice infected with IAV 7 days after SP had 0% mortality, while other groups suffered from mortality from 25% to 100% [19]. Deprivation of commensal bacteria (such as SP and SA) from respiratory tract exacerbated influenza-induced disorder [123–125]. Recently, an* in vivo* experiment showed that preceding SP infection protected mice from IAV-related detriment [126]. One of possible mechanisms underlying this virus antagonistic effect is that bacteria create an inflammatory environment [40]. Indeed, SP infection promoted IFN-*γ* production [127–131], which results in an antiviral state (Figure 1(b)). However, the role of autophagy and apoptosis in this phenomenon is paid less attention.

#### 2.2.2. Role of Autophagy in Postbacterial IAV Infection

Autophagy seems to play a critical role in improving host immunity in SP infection (Figure 1(b)). Guo et al. found that SP-induced autophagy was a defense mechanism against bacterial infection [132]. The mechanism may be partly attributed to recognition of LPS of SP by TLR4 to trigger autophagy through receptor-interacting protein 1 (RIP1-P38) signaling to promote SP clearance [133, 134]. Also, Li et al. reported that SP clearance was enhanced by autophagy; conversely bacterial clearance was reduced by autophagy suppression [135]. In addition, although preadministrated SP failed to produce any detectable effects on either cell morphology or IAV replication in epithelial cells [136], another* in vivo* study conducted by Wang et al. showed bacteria colonization, to some extent, indeed preventing viral infection [125]. Comparing to SA-free mice and wild-type mice, specific pathogen-free (SPF) mice were more susceptible to fatalness induced by IAV [125]. This is consistent with the results from McCullers and Rehg, although different cocci were used [19]. One of possible mechanisms underlying this result is that peripheral CCR2+CD11b+ monocytes were recruited into alveoli and then were polarized to M2 alveolar macrophages by the interaction between SA and TLR2. Therefore, SA colonization increases immunity ability. Nonetheless, as TLR2 is crucial for lung homeostasis rather than bacterial elimination, TLR2 deficiency failed to interfere in SA elimination [125]. TLR2 was also reported to trigger autophagy through JNK signaling [137]. In this situation, TLR2 could serve as a stimulus for the development of bacteria-induced autophagy. These may explain why Ouyang et al. did not detect the influence of SP pretreatment on IAV replication. Recently, Wolf et al. reported that pneumolysin, a bacterial virulence factor important in inducing immune responses, protected postpneumococcus IAV infection in an* in vivo* model [126]. Li et al. further supported that pneumolysin was a key factor in triggering autophagy through ROS hypergeneration and inhibition of PI3K-I/Akt/mTOR pathways in A549 cells [135]. In other words, SP might exert protective effects against IAV via autophagy mechanism. Taken together, it is clear that preceding SP infection produces systematic defense reactions, including autophagy to attenuate the followed IAV infection.

## 3. Role of Autophagy and Apoptosis in Viral-Bacterial Coinfection: A Potential Research Field

### 3.1. Present Treatment against IAV and Bacterial Infection

Present treatments against IAV and bacterial infection include viral and bacterial vaccines, antiflu drugs, and antibiotics. As reviewed by Christopoulou et al., application of viral and bacterial vaccines effectively decreases post-IAV bacterial infection [138]. Antiflu drugs are also shown to not only reduce IAV infection but also decrease clinical morbidity of secondary bacterial infection [139, 140]. Nonetheless, as IAV is an ssRNA virus with 8 segments, which contribute to low self-correcting ability during transcription, IAVs are easily to produce new mutants which are consequently resistant to antiviral drugs or fail to be neutralized by vaccines [42, 141, 142]. Additionally, multidrug resistant SA (MDRSA), especially methicillin-resistant SA (MRSA), have been widely disseminated in hospital and community [143, 144]. For these concerns, although vaccines and antibiotics are currently primary treatments for possible post-IAV bacterial infections, further mechanism exploration for more treatment targets, including autophagy and apoptosis, is still significantly important.

### 3.2. Autophagy and Apoptosis Regulation: A Potential Alternative Method to Fight against Increased Susceptibility to Post-IAV Bacterial Infection

Here, we propose an assumption that regulating autophagy at the early stage or suppressing apoptosis at the late stage may be a promising strategy to antagonize post-IAV SP pneumonia. Until now, there are a number of studies showing that autophagy suppression ameliorates the impact of IAV risk [45, 51, 84–91]. Although IAV has been proved to inhibit autophagy to facilitate its replication, decreased viral titer was also observed in presence of autophagy stimulator rapamycin in MDCK cells [48]. What is more, autophagy plays a protective role in adaptive immunity against virus-infected hosts [81, 82]. Some chemicals exert virus suppressive effect by triggering autophagy [83, 84]. These indicate that excessive or insufficient autophagy is detrimental for IAV.

What needs to be paid special attention is a moderate regulation of autophagy. Hahn et al. established a mouse model in which Atg5 gene was knockout in the distal respiratory epithelium to achieve different degrees of autophagic reduction [145]. They infected these mice with 50% autophagy ability with H3N2 virus. The results showed that viral replication was decreased, lung structure and function were improved, and morbidity and mortality were decreased. When mice with 10% autophagy ability were used, lung injury in elderly group was exacerbated with time, while alveolar septum was thickened in adult group. Therefore, an appropriate autophagic level is necessary to fight against IAV invasion. As aforementioned, inhibiting apoptosis presents an overall beneficial effect for hosts infected by IAV [118–121]. Thus, treating IAV infection with autophagy regulators and apoptosis inhibitors in* in vivo* models can be regarded as a potential researching field to explore a new breakthrough fighting against influenza and its sequelae.

The significance of this assumption is that the high conservativeness of autophagy and apoptosis in eukaryotes, to some extent, prevents the complexity of IAV mutation. The concept that autophagy and apoptosis are conserved among eukaryotes has been applied in drug screening and discovery, where cell-based assays were used for antiviral drug screening [86, 87, 89]. Further validation is necessary in* in vivo* models.

## 4. Conclusion

Collectively, IAV facilitates its host to suffer from bacterial pneumonia via various pathways. Among the underlying mechanisms, autophagy and apoptosis act as sequential events to regulate post-IAV bacterial pneumonia. Systemic analysis of autophagy and apoptosis may provide a new strategy for prophylactic and therapeutic treatment of influenza virus infection.
